# The Secretome Derived From Mesenchymal Stromal Cells Cultured in a Xeno-Free Medium Promotes Human Cartilage Recovery *in vitro*

**DOI:** 10.3389/fbioe.2020.00090

**Published:** 2020-02-14

**Authors:** Maria Elisabetta Federica Palamà, Georgina Margaret Shaw, Simonetta Carluccio, Daniele Reverberi, Laura Sercia, Luana Persano, Dario Pisignano, Katia Cortese, Francis Peter Barry, Josephine Mary Murphy, Chiara Gentili

**Affiliations:** ^1^Laboratory of Cellular Oncology, Department of Experimental Medicine, University of Genoa, Genoa, Italy; ^2^Regenerative Medicine Institute, National University of Ireland Galway, Galway, Ireland; ^3^U.O. Molecular Pathology, IRCCS Ospedale Policlinico San Martino, Genoa, Italy; ^4^Institute of Nanoscience (CNR-NANO), Pisa, Italy; ^5^Department of Physics, University of Pisa, Pisa, Italy; ^6^Department of Experimental Medicine, Human Anatomy, University of Genoa, Genoa, Italy

**Keywords:** mesenchymal stromal cells, secretome, extracellular vesicles, xeno-free medium, inflammation, osteoarthritis

## Abstract

Osteoarthritis (OA) is a disabling joint disorder causing articular cartilage degeneration. Currently, the treatments are mainly aimed to pain and symptoms relief, rather than disease amelioration. Human bone marrow stromal cells (hBMSCs) have emerged as a promising paracrine mechanism-based tool for OA treatment. Here, we investigate the therapeutic potential of conditioned media (CM) and extracellular vesicles (EVs) isolated from hBMSC and grown in a xeno-free culture system (XFS) compared to the conventional fetal bovine serum-culture system (FBS) in an *in vitro* model of OA. First, we observed that XFS promoted growth and viability of hBMSCs compared to FBS-containing medium while preserving their typical phenotype. The biological effects of the CM derived from hBMSC cultivated in XFS- and FBS-based medium were tested on IL-1α treated human chondrocytes, to mimic the OA enviroment. Treatment with CM derived from XFS-cultured hBMSC inhibited IL-1α-induced expression of IL-6, IL-8, and COX-2 by hACs compared to FBS-based condition. Furthermore, we observed that hBMSCs grown in XFS produced a higher amount of EVs compared to FBS-culture. The hBMSC-EVs not only inhibit the adverse effects of IL-1α-induced inflammation, but play a significant *in vitro* chondroprotective effect. In conclusion, the XFS medium was found to be suitable for isolation and expansion of hBMSCs with increased safety profile and intended for ready-to-use clinical therapies.

## Introduction

Articular cartilage is a specialized connective tissue consisting of chondrocytes embedded in a defined and highly organized matrix (Zhang, 2015). This structure confers relevant mechanical properties to the tissue, including load-bearing capacity (Roughley, 2006; Responte et al., 2007). Nevertheless, the matrix composition changes with aging and cartilage may progressively lose its biomechanical function. Bone and joint injures may also lead to development of osteoarthritis (OA) (Cancedda et al., 2003), since articular cartilage has limited self-regeneration capacity (Responte et al., 2007). OA, as the primary degenerative joint disease, affects approximately 250 million people worldwide and causes disability especially in older populations (Toh et al., 2016). Pathological alterations in OA include articular cartilage degradation, degeneration of menisci and ligaments and thickening of the subchondral bone (Loeser et al., 2012). Although the exact pathogenesis hasn’t been fully elucidated, OA is associated with a local inflammatory responses (Feldmann, 2001). In diseased cartilage, chondrocytes become hypertrophic and start to synthesize a matrix inappropriate for its primary weight-bearing function (Goldring and Goldring, 2010). This increase in metabolic activity in response to cartilage injury leads to matrix changes associated with tissue degeneration rather than regeneration (Mort and Billington, 2001).

It has been reported that inflammatory cytokines such as prostaglandins, tumor necrosis factor (TNF)-α, interleukin (IL)-1, IL-6 and nitric oxide are involved in the progression of OA. These factors have also been described as powerful activators of cartilage damage in *in vitro* models, acting through NF-kB pathway activation and production of cyclooxygenase-2 (COX-2) (Ulivi et al., 2008; Pereira et al., 2013). The role of COX-2 is pivotal both *in vitro* and *in vivo* inducing the production of eicosanoids, which in turn affect the metabolism of chondrocytes to create an imbalance toward catabolic activity (Pelletier et al., 2001). Thus, the NF-kB pathway promotes the destruction of the articular joint during OA development by triggering the expression of an array of genes including cytokines, chemokines, immunoreceptors, cell adhesion molecules, acute phase proteins and stress response genes.

Among current pharmacological treatments for OA, non-steroidal anti-inflammatory drugs, analgesics and corticosteroids aim to provide pain relief and control of inflammation (Hunter, 2011), but have a low impact in preventing disease progression. BMSCs have emerged as a promising biological therapeutic option for OA (Murphy et al., 2003; Ruiz et al., 2016; de Windt et al., 2017) thanks to their distinctive immunomodulatory and regenerative features (Uccelli and de Rosbo, 2015). Nevertheless, the molecular mechanism underlying the beneficial effects in cartilage repair remains elusive. In the last decade, a new hypothesis has emerged proposing that stem cells exert their therapeutic effects not through their tissue engraftment and replacement of resident cells, but rather *via* secretion of soluble factors and extracellular vesicles (EVs) to support endogenous restoration. In fact, BMSCs and all types of stem cells secrete EVs, which have been shown to have regenerative potential both *in vitro* and *in vivo* (von Bahr et al., 2012; Gnecchi et al., 2016).

Extracellular vesicles are cell-derived membranous structures surrounded by a phospholipidic bilayer containing signaling molecules, including proteins, lipids, DNA fragments, RNA and miRNA. Among various subgroups of EVs, microvesicles generally refer to 150–1,000 nm bodies released by budding from the plasma membrane (PM). The term exosome indicates 30–100 nm vesicles of endosomal origin released following the fusion of multivesicular bodies with the PM (Johnstone et al., 1987).

Despite the extensive research done in the field of EVs as therapeutic tools, there are still many issues to be addressed, especially those related to safety, isolation and cell culture methodology. Most cell cultures are carried out in media containing fetal bovine serum (FBS), which could represent a source of contamination. Moreover, cellular products intended for use in clinical therapy must be completely free of animal derivatives as required by safety issues. In this context, the use of human serum or platelet derivatives has been proposed as a substitute for FBS. Although these products have not risk of immunoreactivity, problems of donor variability still remain. In this scenario, it seems crucial to develop a xeno-free culture system for the generation of clinical-grade MSC-secretome.

In our study, we used a novel xeno-free supplement (XFS) for isolation and expansion of hBMSC in comparison with conventional FBS-based culture. We characterized the cells and investigated the therapeutic anti-inflammatory potential of the secreted conditioned media (CM) and EVs produced in both aforementioned culture conditions on primary cultures of human articular chondrocytes (hACs) using an *in vitro* inflammatory model (Pereira et al., 2013).

Accordingly, we trigger inflammation by treating hACs with IL-1α, alone or in combination with CM or EVs derived from hBMSCs grown in FBS- or XFS-containing medium. To elucidate the role of hBMSC-derived products, we investigated the activation of the NF-kB pathways and the expression of COX-2, IL-6, and IL-8 in hACs for each treatment.

## Materials and Methods

### Primary Cell Cultures

Human bone marrow stromal cells were derived from hip bone marrow aspirates of healthy donors using a protocol approved by the Clinical Research Ethical Committee at University College Hospital, Galway, Ireland after informed consent. The marrow was processed at the National University of Ireland Galway (NUIG) for isolation of the cells. Briefly, the nucleated cell fraction of the bone marrow aspirate was suspended in α-MEM-GlutaMAX medium (GiBCo, Waltham, MA, United States), supplemented with either: (i) 10% FBS (Sigma-Aldrich, St Louis, MO, United States), 1 ng/ml Fibroblast Growth Factor-2 (FGF-2, Peprotech, London, United Kingdom) and 100 U/ml penicillin-100 μg/ml streptomycin mixture (GiBCo) for standard culture conditions, (FBS-hBMSCs) or (ii) XFS (patent No. PCT/EP2015/053223) (Barry et al., 2015) and 100 U/ml penicillin-100 μg/ml streptomycin mixture (XFS-hBMSCs). The cells were plated at a density of 2.3 × 10^5^ cells/cm^2^. After 5 days, the flasks were washed with Dulbecco’s Phosphate Buffer Saline (D-PBS) to remove unattached cells and fresh medium added. When large distinct colonies were formed, hBMSCs were detached with a TrypLE Express 1X disassociation solution (GiBCo) and plated at a density of 3,000 cells/cm^2^. Cell cultures were maintained in an incubator at 37°C with 5% CO_2_ and medium was changed every 2 days. A total number of six primary cultures were analyzed.

Human articular chondrocytes were obtained from femoral condyles of patients undergoing partial knee arthroplasty, after informed consent and under the approval of the Ethical Committee of San Martino Hospital (Genoa, Italy). Articular cartilage was separated from subchondral bone and fragmented. Tissue pieces were subjected to repeated enzymatic digestions in 1–2 ml mixture consisting of 1 mg/ml hyaluronidase (Sigma-Aldrich, Saint Louis, MO, United States), 400 U/ml collagenase I (Worthington Biochemical, Lakewood, NJ, United States), 1000 U/ml collagenase II (Worthington Biochemical) and 0.25% trypsin (GiBCo) in Dulbecco’s Modified Essential Medium (DMEM) High Glucose (EuroClone). The isolated hACs were seeded in 1–2 wells of a 6-well plate in DMEM High Glucose with 10% FBS, 2 mM L-glutamine (EuroClone), 100 U/ml penicillin-100 μg/ml streptomycin, 1 mM Na-pyruvate (EuroClone) and 10 mM HEPES (EuroClone). The medium was changed every 2–3 days. Cell cultures were maintained in an incubator at 37°C with 5% CO_2_. At ∼90% of confluence, cells were detached and plated at different densities.

### Growth Kinetics and Phenotypic Characterization of hBMSCs

A growth curve was generated for both cultured hBMSCs in α-MEM-GlutaMAX supplemented with 10% FBS or with XFS. At each passage, cells were detached with TrypLE Express 1X disassociation solution, counted and re-plated at the same density (∼3,000 cells/cm^2^). Cell cultures were maintained for 7–8 passages (∼30 days). Cumulative cell doublings were plotted against time to determine the growth kinetics. This experiment was performed for a total of six primary cultures. Cell morphology was monitored using optical microscopic observation.

For phenotypic characterization by flow cytometry 100,000 cells cultured with either 10% FBS or XFS, were incubated with 1 μl of antibodies conjugated with fluorescein isothiocyanate (FITC) or phycoerythrin (PE): CD31-FITC, CD34-FITC, CD45-PE, CD73-FITC, CD105-PE, CD146-FITC, (Biosciences, San Diego, CA, United States), CD90-PE, CD166-PE, CD106-PE, HLA-ABC-PE, HLA-DR-FITC (BD Pharmingen San Diego, CA, United States). The appropriate IgG-isotype controls conjugated with FITC or PE (Bioscience) were also used to stain the cells. The staining was performed for 30 min at 4°C in the dark and samples were run on a Cyan ADP cytofluorimeter (Beckman-Coulter, Brea, CA, United States). Data were analyzed using FlowJo_V10 software (BD BioSciences, Franklin Lakes, NJ, United States) and expressed as Log fluorescence intensity vs. number of cells. Analysis was performed on six different primary cultures at passage 2.

### Propidium Iodide (PI) and FITC-Anti-annexin-V Apoptosis Detection

To detect apoptosis, hBMSCs cultured in FBS- or XFS-containing media were analyzed using flow cytometry, at passage 2. Double staining for Annexin-V and Propidium Iodide (PI) was performed to determine if cells were viable, early apoptotic or apoptotic using the Annexin-V apoptosis assay kit (BD Pharmingen, San Diego, CA, United States), according to the manufacturer’s instructions.

### Preparation of Conditioned Medium (CM) From hBMSCs

Human bone marrow stromal cells were cultured until ∼70% of confluence in FBS- or XFS-containing medium. The medium was discarded and, after 3 washes with D-PBS, it was replaced with a basal medium (α-MEM-GlutaMAX supplemented with 100 U/ml penicillin-100 μg/ml streptomycin). Cells were incubated (5% CO_2_ at 37°C) for 24 h (for whole secretome analysis) or 72 h (for EV isolation). The hBMSCs-CM was collected and processed as described below.

### Isolation of EVs From hBMSCs (hBMSC-EVs)

hBMSCs-EVs were obtained after high-speed differential centrifugation of hBMSCs-CM collected from 10 × 10^6^ hBMSCs cultured to 70% confluence in either FBS- or XFS-containing media and thereafter cultured in basal medium for 72 h. The FBS was depleted from FBS-derived extracellular vesicles by ultracentrifugation at 100,000 *g* over night at 4°C. hBMSCs-CM collected from monolayer culture were centrifuged at 300 *g* for 10 min at 4°C to eliminate dead cells and debris. Once the supernatant was recovered, a second centrifugation at 2,000 *g* for 20 min at 4°C was carried out to eliminate apoptotic bodies from the preparation. The supernatant was transferred to ultracentrifuge polyallomer tubes (Beckman-Coulter) for ultracentrifugation at 10,000 *g* for 30 min at 4°C to pellet microvesicles and larger vesicles. A second ultracentrifugation at 100,000 *g* for 90 min at 4°C was used to pellet the exosomes and smaller vesicles. The pellet was washed with D-PBS, previously filtered through 0.22 μm filter to remove any particles in the solution. The cell monolayers were trypsinized and counted.

All EV pellets were resuspended into a single clean tube and a final ultracentrifugation was carried out at 100,000 *g* for 70 min at 4°C to wash the isolated vesicles. The supernatant was recovered and kept as conditioned medium depleted from EVs (CM^–^). The pellet was resuspended in 100 μl of filtered D-PBS, adding 2 mM EDTA to prevent the EVs clustering. A Beckman-Coulter ultracentrifuge (Beckman-Coulter Optima XPN-100 ultracentrifuge, Beckman-Coulter) was used with swinging bucket rotors type SW28 and SW41Ti.

### Analysis of Cytokine Content in CM and EVs From hBMSCs

For analysis of whole CM, hBMSCs-CM was collected after 24 h in basal medium, and immediately centrifuged at 300 *g* for 10 min at 4°C to remove dead cells and the supernatant was centrifuged again at 2,000 *g* for 20 min to remove debris and apoptotic bodies. The CM were concentrated with Amicon Ultra filters with 3 KDa molecular weight cut-off (Merck, Darmstadt, Germany). For analysis of isolated EVs, hBMSCs-CM was collected after 72 h in basal medium and EVs were isolated as described before and the EV pellet was resuspended in RIPA buffer (1% Non-idet p-40, 0.1% sodium deoxycholate, 0.1% sodium dodecyl sulfate in PBS pH 7.5) freshly supplemented with a protease inhibitor cocktail (Sigma-Aldrich). Protein content was quantified by Bradford assay (for CM, SERVA, Heidelberg, Germany) or BCA assay (for EVs) and 10 μg protein were analyzed with a Proteome Profiler Human XL Cytokine Array Kit (R&D, Minneapolis, MN, United States) following the manufacturer’s instructions. The membranes were scanned using the Epson Perfection 1260 scanner (Epson, Suwa, Japan) and spot densities were quantified using Fiji-ImageJ software (U. S. National Institutes of Health, Bethesda, MD, United States). Gene Ontology (GO) was also carried out using DAVID (Functional Annotation Bioinformatics Microarray Analysis). This analysis was performed on two different primary cultures (each one in duplicate) at passage 2 for whole CM and on a pool derived from three different primaries for EVs.

### Characterization of hBMSC-EVs

The concentration of membrane-bound protein on the surface of freshly isolated, intact hBMSC-EVs was measured using the BiCinchoninic Acid (BCA) assay (Thermo Scientific Pierce, Rockford, IL, United States) following the manufacturer’s instructions. The total protein content was normalized to the number of FBS-hBMSCs and XFS-hBMSCs (1 × 10^6^ cells). Six different preparations were analyzed for each condition.

At least three independent preparations of both FBS-hBMSC-EVs and XFS-hBMSC-EVs were stained with 1 μM Cell Trace Far Red (ThermoFisher Scientific, Waltham, MA, United States) in combination with the mouse anti-human monoclonal antibody (mAb) PE-Cy7-CD63 (a vesicular marker; Clone: H5C6 BD Pharmingen, San Jose, CA, United States) or the anti-human mAb PE-CD105 (a MSC marker; Clone: SN6 eBioscience, San Diego, CA, United States), both diluted 1:100 in filtered D-PBS. A set of microsphere suspensions (1.35 and 0.88 μm, SpheroTec, Martinsried, bei München, Germany) was used as a size reference. An unstained sample was acquired to detect the sample autofluorescence and set the photomultiplier for the three used channels. Forward and side scatter channels (FSC-A and SSC-A) were used on a logarithmic scale visualized in bi-exponential mode. The FSC and SSC photomultipliers were set using background noise as the lower optical limit using a tube containing sterile, filtered D-PBS. The threshold, set on the FSC-A channel, was regulated to reduce the noise progressively, allocating dots in the lower left corner of the plot, in order to clearly detect EVs. Samples were run on a FACS Aria II (BD Biosciences, San Jose, CA, United States) cytofluorimeter. At least 2 × 10^4^ events were analyzed in each instance.

The concentration (i.e. number of particles/ml for a specified size range), particle size and number-based size distribution of EV samples derived from hBMSCs grown in FBS- or XFS-containing medium was defined using Tunable Resistive Pulse Sensing (TRPS). Analysis was performed on FBS-hBMSC-EVs and XFS-hBMSC-EVs derived from three different primary cultures using qNano (Izon Science Ltd., Burnside, Christchurch, New Zealand). The particle concentration was normalized to 1 × 10^6^ cells and the protein content to define the particle/protein ratio.

### Analysis of Pre-conditioned Cells by Scanning Electron Microscopy

Human bone marrow stromal cell were cultured in either FBS- or XFS- containing media on cover glasses at a density of 3,000 cell/cm^2^ and then pre-conditioned for EV production as described. Cells were washed with D-PBS and fixed with 2.5% glutaraldehyde (Sigma-Aldrich) for 30 min at room temperature, then rinsed twice in cacodylate buffer solution (Na-cacodylate 1 mM in PBS). Lipid fixation with osmium tetroxide (OsO_4_, Sigma-Aldrich) was performed to better discriminate membranes and vesicles; cells were incubated for 1 h with 0.1% OsO_4_ and washed at least three times with cacodylate buffer to remove any trace of osmium. The samples were then dehydrated in increasing concentrations of ethanol (50, 70, 95 and 100%), transferred to an increasing graded series of hexamethyl–disilazane (HMDS, 50% in ethanol and 100%) and left to dry overnight. Samples were then covered with a 7 nm chromium-film and 20 nm gold-film using a thermal evaporator (Sistec). The morphology of cells and EVs was evaluated using SEM operating at an acceleration voltage of 5 kV (MERLIN^TM^ – Field Emission Scanning Electron Microscope, Carl Zeiss).

### Analysis of EV Preparations by Transmission Electron Microscopy

Electron microscopic analysis was performed on isolated vesicle preparations as follows. The pellets were resuspended in 20 μL filtered D-PBS (pH 7.4) and fixed by adding an equal volume of 2% paraformaldehyde in 0.1 mol/L phosphate buffer (pH 7.4). EV preparations were first adsorbed for 10 min to formvar-carbon coated copper grids by floating the grids on 5 μl drops on parafilm. Grids with adhered vesicles were then rinsed in D-PBS and negatively stained with 2% uranyl acetate for 5 min at room temperature. Subsequently, grids were embedded in 2.5% methylcellulose for improved preservation and air dried before examination. Electron micrographs were taken at Hitachi TEM microscope (HT7800 series, Tokyo, Japan) equipped with Megaview 3 digital camera and Radius software (EMSIS, Germany).

### Labeling and Internalization of hBMSC-EVs by OA hACs

Extracellular vesicle uptake was monitored *in vitro* by staining FBS-hBMSC-EVs and XFS-hBMSC-EVs with PKH67 (Sigma-Aldrich), according to the manufacture’s protocol. Staining was stopped by adding an equal volume of 1% bovine serum albumin and the EVs were ultracentrifuged to remove unbound dye. Chondrocytes (OA hACs), plated on cell culture slides were treated with 200 U/ml IL-1α (Peprotech, London, United Kingdom) overnight to mimic an inflammatory condition *in vitro* or left in normal culture condition as a control. After pre-treatment, 1 μg/ml of stained FBS- or XFS-hBMSC-EVs was added to the cells for 3 h prior to fixation with 3.7% paraformaldehyde (PFA) and staining with 5 U/ml phalloidin-594 (Life-Tech) to visualize the cytoskeleton. Nuclei were stained with DAPI (Sigma-Aldrich) and coverslipped with an aqueous mounting. Slides were observed at different magnifications and images acquired with an Axiovert 200M microscope (Carl Zeiss Microscopy GmbH, Jena, Germany). Parallel controls with completed and depleted CM were also carried out in each experiment (*n* = 5).

### Western Blotting

Confluent hACs (passage 1) were treated for 16 or 48 h with 200 U/ml IL-1α ± FBS-hBMSC-CM or XFS-hBMSC-CM at 1, 10, and 100 μg/ml. HAC-CM was collected and 200 μl of each sample transferred to 1.5 ml tubes. Proteins were precipitated using 10% trichloroacetic acid (TCA) and centrifuged for 20 min at 14,000 rpm. Protein pellets were resuspended in NuPAGE Sample Buffer (Life-Tech) and loaded on 4–12% NuPAGE Bis-Tris gels (Life-Tech). Electrophoresis and blotting were performed as described previously (Pereira et al., 2013). Briefly, nitrocellulose membranes (GE Healthcare Life Sciences, Freiburg, Germany) were incubated overnight at 4°C with specific primary antibodies against IL-6 and IL-8 (1:200, Santa Cruz Biotechnology, Santa Cruz, CA, United States). After washing, membranes were exposed to horseradish peroxidase-linked goat anti-mouse IgG for 1 h at room temperature (1:5,000, GE Healthcare) and bands were visualized using enhanced chemiluminescence (GE Healthcare). X-ray films (Fujifilm GmbH, Düsseldorf, Germany) were exposed to the membranes, developed and fixed prior to scanning using the Epson perfection 1260 scanner with band densities quantified using Fiji-ImageJ software.

Western blot analysis for COX-2 expression in cell lysates was also performed. OA hACs were scraped into 30 μl of RIPA buffer and protein concentration quantified by Bradford assay. Protein (10 μg of each sample) was loaded on 4–12% NuPAGE Bis-Tris gels for electrophoresis prior to transfer to nitrocellulose membranes. Blotted membranes were incubated with a specific primary antibody against COX-2 (1:500, Abcam, Cambridge, United Kingdom).

To investigate the effect of hBMSC-EVs, confluent hACs (at passage 1) were treated for 16 and 48 h with 200 U/ml IL-1α ± 1 μg/ml FBS-hBMSC-EVs, 1 μg/ml XFS-hBMSC-EVs or with a corresponding dose of complete CM (CM^+^) and depleted CM (CM deprived from EVs, CM^–^). To correctly compare the different conditions, a normalization based on number of cells was considered. In a parallel experiment hACs were pre-treated for 3 h with 1 μg/ml FBS-hBMSC-EVs, 1 μg/ml XFS-hBMSC-EVs, FBS/XFS-CM^+^ or FBS/XFS-CM^–^ and then treated with 200 U/ml IL-1α prior to Western blot analysis. A negative control without IL-1α or EVs and a positive control treated only with 200 U/ml IL-1α were included in all the experiments and all analyses were performed on a minimum of six different primary hAC preparations.

### NF-kB Nuclear Translocation

HACs from six different donors were cultured in 24-well plates on glass coverslips (10,000 cells) and treated for 4 or 40 h with a medium supplemented with 200 U/ml IL-1α ± FBS-hBMSC-CM or XFS-hBMSC-CM at three different concentrations 1, 10, and 100 μg/ml, following an *in vitro* model well described by our group (Pereira et al., 2013). Cells were fixed in 3.7% PFA for 10 min at room temperature. After washing in D-PBS, cells were permeabilized with a solution containing 20 mM HEPES, 300 mM sucrose, 50 mM sodium chloride, 3 mM magnesium chloride, and 0.5% Triton X-100 for 10 min at 4°C. Non-specific binding was prevented by incubation with 20% goat serum (EuroClone) in PBS for 30 min at 4°C. Slides were incubated with a rabbit anti-NF-kB p65 antibody (1:400; Cell Signaling, Danvers, MA, United States) overnight at 4°C. After rinsing in D-PBS, a goat anti-rabbit fluorescence-labeled antibody Alexa fluor-488 (Life Technologies, Grand Island, NY, United States) was added for 30 min at 4°C. Cells were stained with 5 U/ml phalloidin-594 for 20 min to visualize the cytoskeleton. Coverslips were mounted with an aqueous mounting. Slides were observed at different magnifications and images acquired with the Axiovert 200M microscope. A quantification was also performed counting cell with positive nucleus on at least five different areas for each experiment.

### Statistical Analysis

All data are shown as mean ± standard deviation (SD). Data were analyzed with GraphPad Prism^®^ 8.0 software (GraphPad Software, Inc., San Diego, CA, United States). Normal distribution of values was assessed by the Shapiro–Wilk normality test. Statistical analysis was performed using unpaired Student’s *t*-test for normally distributed data (proliferation, surface marker expression, apoptosis, BCA, TRPS, FACS), and Mann–Whitney test in the absence of a normal distribution (cytokine array on CM). One-way ANOVA followed by Tukey’s multiple comparison test was used for western blot analysis and two-way ANOVA followed by Tukey’s multiple comparison test for NF-kB nuclear translocation and PKH67 staining. Level of significance was set at *p* < 0.05 (^∗^*p* < 0.05, ^∗∗^*p* < 0.01, ^∗∗∗^*p* < 0.001). The number of data used for the statistical analyses is indicated in the figure legends and corresponds to independent experiments.

## Results

### hBMSCs Grown *in vitro* in FBS- or XFS-Containing Medium Showed Different Growth Kinetics and Morphology but a Similar Phenotypic Profile

**FIGURE 1:**
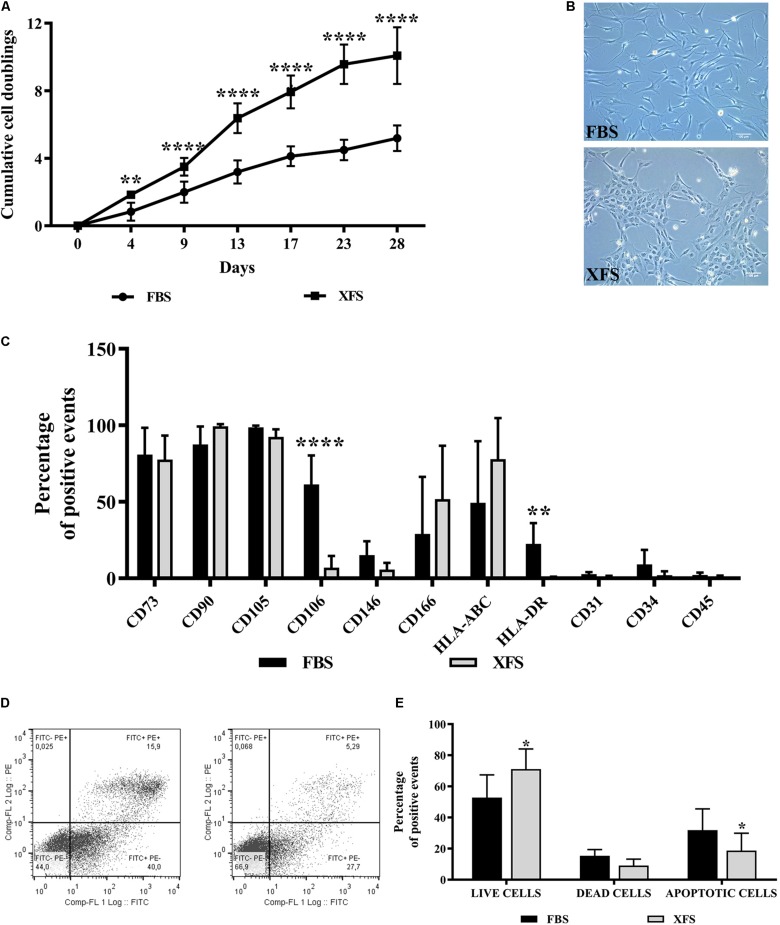
Characterization of hBMSCs cultured in FBS- or XFS-containing medium. **(A)** Proliferation of hBMSCs grown in FBS or XFS-containing medium (6 donors). Growth kinetics expressed as the cumulative number of cell duplications in relation to the culture time. **P < 0.01, ****P < 0.0001, *t*-test. **(B)** Microscope images showing the morphology of the cells. Images were acquired with a phase contrast Leica DMi1 microscope using a 10× magnification (scale bar 100 μm). **(C)** Flow cytometry analysis of hBMSCs cultured in the presence of FBS- or XFS-containing medium. The average percentage of positive events of each marker is reported in a histogram, indicating the mean ± SD of three independent experiments. **P < 0.01, ****P < 0.0001, *t*-test. **(D)** Detection of apoptosis by concurrent staining with FITC-anti-annexin V and PI of hBMSCs grown in FBS- (left panel) or XFS-containing medium (right panel). **(E)** Quantification of flow cytometry data. The histogram shows the mean ± SD of six independent experiments. *P < 0.05, *t*-test.

Human bone marrow stromal cells obtained from six different donors were analyzed for their proliferation rates. A significant increase of cumulative cell doubling in XFS-hBMSCs was found (10 doublings at 30 days vs. 4–5 in FBS-hBMSCs, Figure 1A). Some differences in morphology were also observed (Figure 1B). Cells cultured in the presence of FBS exhibited a spindle-shaped, fibroblastic-like morphology. In XFS, even in the first passage the cells had a less elongated morphology, appearing to be smaller and less branched (Figure 1B).

A consistent phenotype is also important when isolating and culturing hBMSCs for therapeutic applications. Therefore, we examined the surface phenotype of hBMSCs grown in both culture conditions for two passages. hBMSCs cultured in FBS- or XFS-containing medium showed little variation in the highly expressed markers such as CD73, CD90 and CD105. In agreement with the International Society of Cell Therapy (ISCT) criteria (Dominici et al., 2006), both cultured hBMSC preparations were negative for CD45, CD34 and CD31. The markers with the largest differences between different culture conditions were CD106 and HLA-DR, which appeared down-regulated in XFS cultures (Figure 1C).

To exclude any negative effect on cell viability, the number of apoptotic cells in the cultures was assessed with a PI/FITC-anti-annexin-V staining. Differences were observed between the cell populations grown in FBS or XFS, with a significant increase of live cells (71.1 ± 12.9 cells vs. 52.8 ± 14.5 cells in FBS) and a decrease of apoptotic cells (18.7 ± 11.1 cells vs. 31.8 ± 13 cells in FBS) in XFS samples (Figure 1D).

### Secretome Profile of hBMSCs: Cells Grown in XFS Showed Expression of Several Cytokines Involved in Homeostasis, Immune Response, and Wound Healing

**FIGURE 2:**
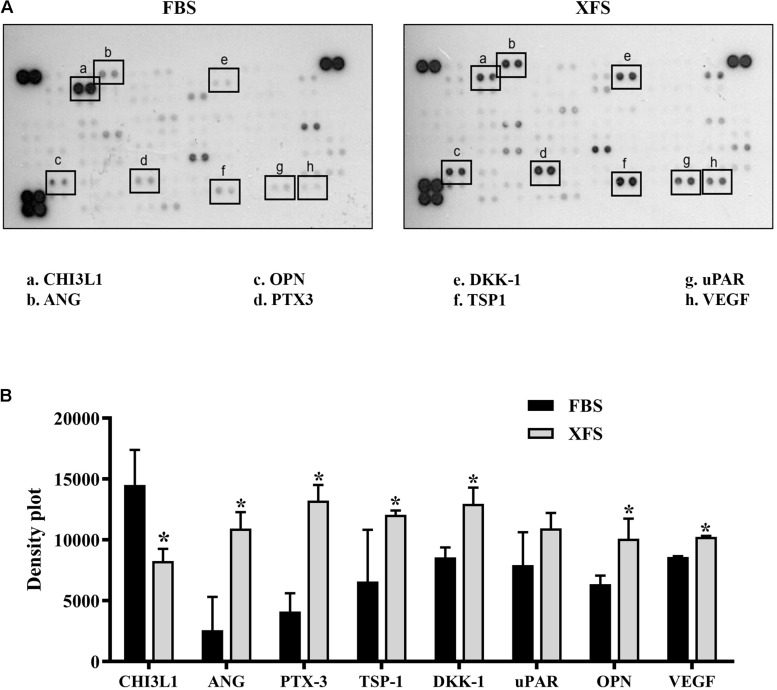
Characterization of secretome protein profile of hBMSCs cultured in FBS- or XFS-containing medium. **(A)** Cytokine array membrane of hBMSCs-CM (FBS-CM and XFS-CM) representative of two independent experiments. **(B)** Quantification of the integrated pixel density for each identified cytokine using Fiji ImageJ. Data are shown as mean ± SD. *P < 0.05, Mann–Whitney. ANG: angiogenin; CHI3L1: chitinase-3-like-1; DKK-1: dickkopf-1 WNT signaling pathway inhibitor; NGAL: lipocalin-2; OPN: osteopontin; PTX-3: pentraxin-3; TSP-1: thrombospondin-1; uPAR: plasminogen activator, urokinase; VEGF: vascular endothelial growth factor.

The analysis of BMSC-CM showed significant differences in the protein content with a down-regulation of Chitinase-3-like-1 (CHI3L1) and a significant up-regulation of several proteins such as angiogenin (ANG), osteopontin (OPN), pentraxin-3 (PTX-3), Dickoff-1 (DKK-1), thrombospondin-1 (TSP-1) and Vascular Endothelial Growth Factor (VEGF) in XFS-CM. A slight, not significant up-regulation of urokinase-type plasminogen activator receptor (uPAR) was also observed (Figures 2A,B).

To better understand the biological roles of these proteins, we performed a Gene Ontology (GO) enrichment analysis with DAVID focusing on the relative frequency of the “biological processes” ontology term (i.e. biological functions) in the proteomic profile of interest (Xu et al., 2017). For both CM we identified several ontologies. Table 1 shows the ontologies related to the proteins modulated by XFS, with the relatives *P*-values. XFS-hBMSCs-CM showed upregulation of proteins influencing immune response (ANG, PTX-3, TSP-1), angiogenesis (VEGF, ANG, TSP-1), homeostatic process (VEGF, OPN, ANG) and wound healing (TSP-1, uPAR, OPN) and downregulation of CHI3L1, which is involved, among other biological processes, in the cell defense response, positive regulation of metabolic processes and phosphorylation.

**TABLE 1:** Gene ontology analysis.

	CHI3L1	ANG	PTX3	TSP-1	DKK-1	uPAR	OPN	VEGF	N°	*P*
Response to external stimulus	*	*	*	*		*	*	*	7	3.3E-6
Defense response	*	*	*	*			*		5	2.8E-4
Positive regulation of metabolic process	*	*		*		*		*	5	5.2E-3
Regulation of phosphorylation	*	*		*	*	*		*	6	1.8E-4
Regulation of response to stress				*		*	*	*	4	1.9E-2
Angiogenesis	*	*		*				*	4	7.7E-4
Endocytosis			*	*	*			*	4	3.1E-3
Secretion	*	*		*				*	4	1.2E-2
Homeostatic process		*					*	*	3	3.7E-2
Immune response		*	*	*					3	3.2E-2
Response to wounding				*		*	*		3	2.1E-3
Response to oxygen levels		*		*				*	3	9.2E-3
Negative regulation of cell death				*		*		*	3	1.1E-1
Cell migration		*		*				*	3	1.1E-1

*Biological processes identified by cytokine clustering from 3 to 7 of the selected cytokines. ANG: angiogenin; CHI3L1: chitinase-3-like-1; DKK-1: dickkopf-1 WNT signaling pathway inhibitor; NGAL: lipocalin-2; OPN: osteopontin; PTX-3: pentraxin-3; TSP-1: thrombospondin-1; uPAR: plasminogen activator, urokinase; VEGF: vascular endothelial growth factor.*

### hBMSC-CM Inhibits IL-1α-Induced Pro-inflammatory Cytokine Secretion and COX-2 Expression in Osteoarthritic Chondrocytes

**FIGURE 3:**
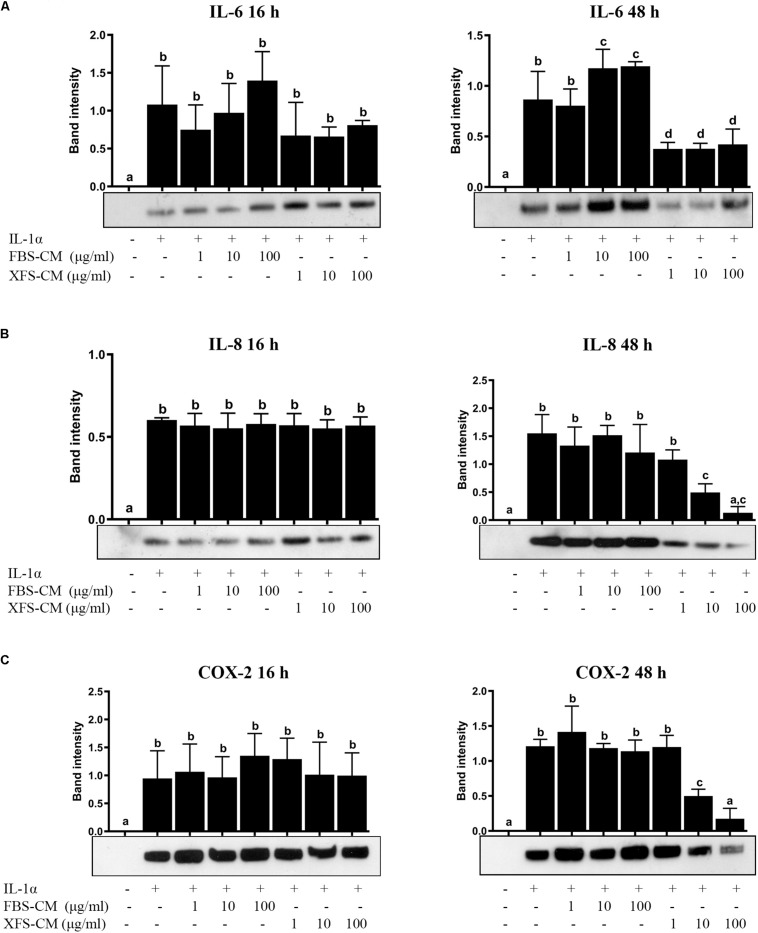
Response of hACs to hBMSC-CM treatment in an inflammatory microenvironment. Western blot analysis of secreted IL-6 **(A**) and IL-8 **(B)** and COX-2 cellular content **(C)** was performed and the corresponding band intensity was determined by densitometric analysis. The intensity values were normalized to tubulin protein expression. Data are shown as mean ± SD and are representative of at least six independent experiments. Values with shared letters are not significantly different (*P* > 0.05) according with one-way ANOVA followed by Tukey as *post hoc* test.

To investigate the effect of hBMSC-CM on OA chondrocytes *in vitro*, we used a model to mimic an inflammatory process, previously described by our group (Pereira et al., 2013). The secretion of IL-6 and IL-8 increased after 16 h when the chondrocyte culture medium was supplemented with IL-1α alone or in combination with FBS-hBMSC-CM or XFS-hBMSC-CM, as compared to controls (Figures 3A,B). After 48 h treatment when hACs were treated with IL-1α alone, IL-6 and IL-8 remained elevated, indicating the persistence of an inflammatory state. Furthermore, when hACs were treated with IL-1α plus FBS-hBMSC-CM at 10 and 100 μg/ml the increase in IL-6 was higher than that elicited by IL-1α alone. After the treatment with IL-1α plus XFS-hBMSC-CM, the levels of both IL-6 and IL-8 were significantly reduced with a concentration-dependent decrease of IL-8 levels.

The effect of hBMSC-CM on COX-2 expression, one of the main effectors of OA in cartilage, was also examined by western blotting. Levels of COX-2 were increased in cells treated with IL-1α, as compared to controls (Figure 3C) after both 16 and 48 h. The simultaneous treatment of hACs with IL-1α and XFS-hBMSC-CM induced an increase of COX-2 after 16 h that was inhibited after 48 h in a dose-dependent fashion, suggesting a role of these CM in resolution of inflammation. Inhibition of COX-2 expression was not found with FBS-hBMSC-CM treatment, where COX-2 levels increased after 16 h and remained high at 48 h.

### hBMSC-CM Inhibits IL-1α-Induced Pro-inflammatory NF-kB Signaling in Osteoarthritic Chondrocytes

**FIGURE 4:**
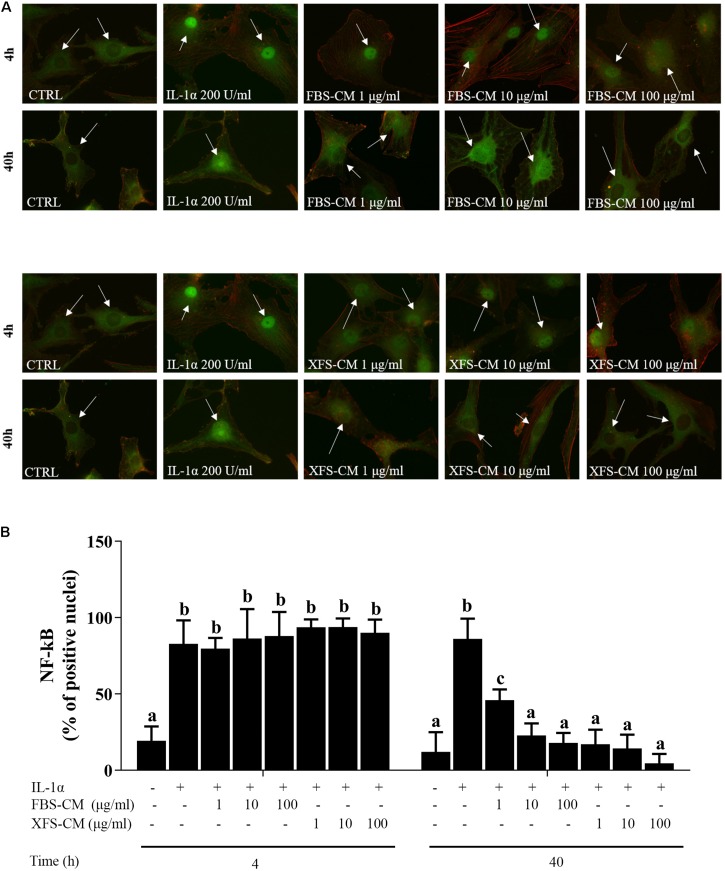
NF-kB nuclear translocation in OA hACs in response to CM treatment. hACs from at least six donors were treated with hBMSC-CM from two allogeneic hBMSC donors and with or without 200 U/ml IL-1α for 4 and 40 h. **(A)** hBMSC-CM inhibited IL-1α-induced nuclear translocation of NF-kB, as showed by immunofluorescence analysis. White arrows show the nucleus of the cells. Representative images of six independent experiments are shown. **(B)** Quantification of the data presented in A. Data are shown as mean ± SD. Values with shared letters are not significantly different (*P* > 0.05) according with two-way ANOVA followed by Tukey as *post hoc* test.

NF-kB signaling is one of the main pathways involved in the initiation and propagation of inflammatory responses (Roman-Blas and Jimenez, 2006). We investigated whether the anti-inflammatory effect of XFS-hBMSC-CM could be mediated through the regulation of NF-kB activity, analyzing its subcellular localization. Treatment of chondrocytes with IL-1α induced translocation of NF-kB from the cytoplasm to the nucleus, after 4 and 40 h suggesting the activation of this pathway (Figure 4A). The increase in NF-kB translocation induced by IL-1α observed at 4 h was not abolished by treatment of the hAC with either FBS-hBMSC-CM or XFS-hBMSC-CM. However, at 40 h both CMs were able to blunt NF-kB activation decreasing its translocation to control levels. XFS-hBMSC-CM seemed to be more effective given that the former significantly reduced NF-kB translocation at all the doses tested, while FBS-hBMSC-CM was not able to downregulate NF-kB signaling at the lowest dose (Figure 4B). These results suggest that both FBS- and SF-hBMSC-CM could potentially affect the OA inflammatory status.

### hBMSCs Grown in Serum-Free Medium Secreted a Higher Amount of EVs

To isolate EVs, hBMSCs were maintained for 72 h in basal medium. Afterward, we assessed cell viability. About 60% viable FBS-hBMSCs were detected, however, XFS-hBMSCs showed a stronger resistance to starvation, exhibiting a proportion of living cells greater than 90% (Supplementary Figure 1).

**FIGURE 5:**
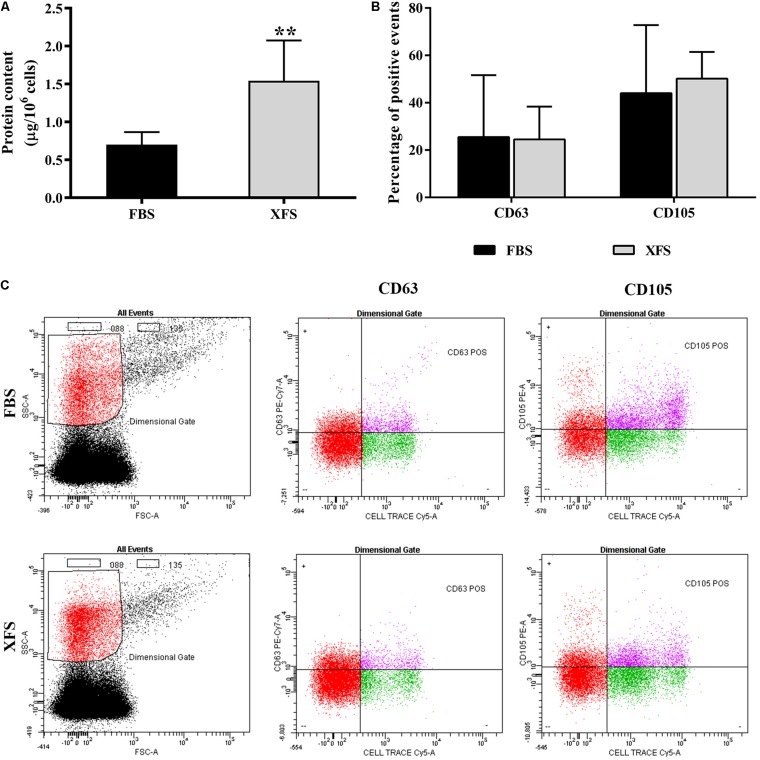
Characterization of EVs derived from hBMSC cultured in FBS- or XFS-containing medium. **(A)** BCA assay of the protein concentration of FBS- and XFS-hBMSC-EVs released by 10^6^ cells. Data are shown as mean ± SD, **P < 0.01, *t*-test and are representative of six independent experiments. **(B)** Histogram showing the percentage of Cell Trace-positive EVs expressing CD63 or CD105. Data are shown as mean ± SD and are representative of three independent experiments. **(C)** Flow cytometry to characterize FBS- and XFS-hBMSC-EVs. Vesicles were stained by the lipophilic dye Cell Trace and specific antibodies against the vesicular marker CD63 or the MSC marker CD105. The left panel shows the physical parameters of samples. The black dots represent the noise and the red ones the dimensional gate, set on the size of reference beads. The right panels show the percentage of Cell-Trace^+^-CD63^+^ or Cell-Trace^+^-CD105^+^ events inside the dimensional gate (pink dots are double positive events).

Extracellular vesicles were then isolated and characterized. Protein content on the surface of EVs was evaluated by BCA assay. A significantly higher amount of proteins was found in the XFS-hBMSC-EVs samples (1.51 ± 0.24/10^6^ cells) respect to the FBS condition (0.54 ± 0.12/10^6^ cells) (Figure 5A), suggesting a higher secretion of EVs by XFS-hBMSCs.

FBS- and XFS-hBMSC-EVs were characterized by flow cytometry using a multiparametric approach. To separate true events from background noise, EVs were defined as events that were included in a dimensional gate which encloses events to 0.88 μm, a process which was established according to well-defined light-scattering profiles of beads with an absolute size (Supplementary Figure 2). EVs were tagged with Cell Trace, an analog of carboxyfluorescein succinimidyl ester (CFSE) to assess vesicular integrity and intact membrane structures. EVs were stained with either the mesenchymal marker CD105 or the vesicular marker CD63. Both FBS-hBMSC-EVs and XFS-hBMSC-EVs expressed the CD105 and CD63 antigens, without significant differences (Figures 5B,C).

**FIGURE 6:**
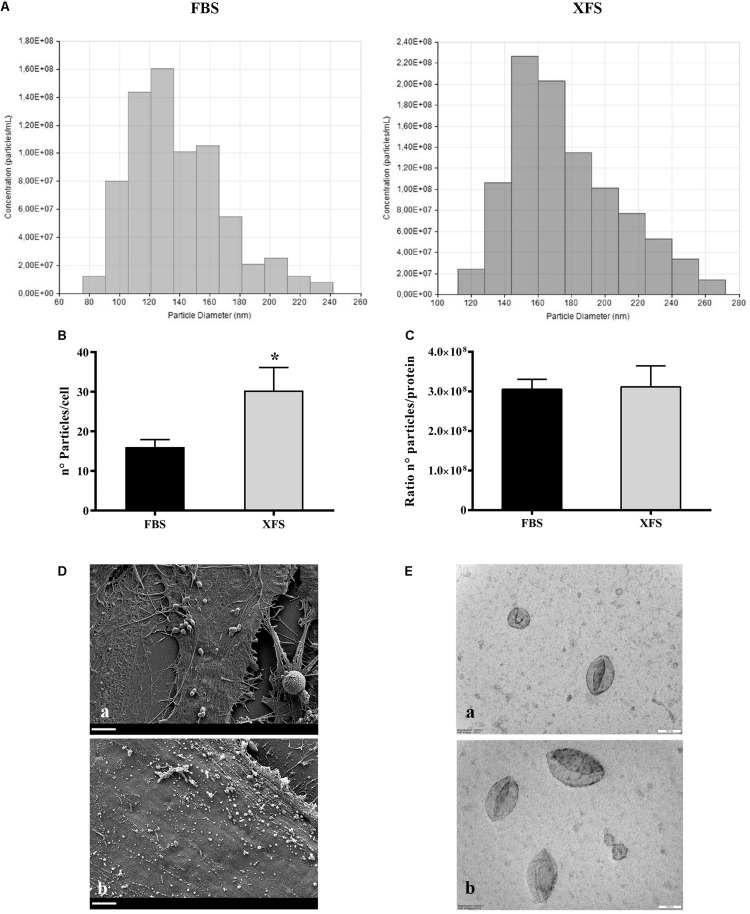
Size distribution and morphological analysis of EVs derived from HBMSCs cultured in FBS- or XFS-containing medium. **(A)** TRPS analysis measuring size distribution of FBS-hBMSC-EVs and XFS-hBMSC-EVs. **(B)** TRPS analysis measuring the number of particles produced by FBS-hBMSCs and -XFS-hBMSCs expressed as number of particles/cell. Data are shown as mean ± SD and are representative of three independent experiments. *P < 0.05, *t*-test. **(C)** Number of particles/protein ratio for a single cell. Data are shown as mean ± SD. **(D)** SEM micrographs of FBS- (a) and XFS-hBMSCs (b) after 72 h of starvation. Scale bar = 2 μm. **(E)** TEM micrographs of FBS- (a) and XFS-hBMSCs-derived (b) isolated EVs. Scale bar = 100 nm.

To estimate the exact particle concentration and the size distribution, FBS-hBMSC-EVs and XFS-hBMSC-EVs were further analyzed via TRPS. Three measurements were run for each sample, in triplicate, and a similar EV size distribution was observed. In both samples, the ultracentrifuged pellet contained hBMSC-EVs between 80 and 300 nm (Figure 6A), thus including both exosomes and microvesicles. Both FBS-hBMSC-EVs and XFS-hBMSC-EVs showed an average size of 160–180 nm. Reasonably high values of particles per cell were found in both conditions, with a two-fold increase in XFS-hBMSCs derived EVs (30.3 ± 5.8 particles/cell) compared to FBS cultured cells (15,0 ± 3.8 particles/cell) (Figure 6B), according also with BCA results. The ratio between the number of particles per cell and protein content was similar in both samples, evidencing a similar protein enrichment and density of proteins on the EV-surface for both FBS- and XFS-hBMSC-EVs (Figure 6C). SEM analysis also confirmed the higher production of vesicles by XFS- hBMSCs compared to FBS-hBMSCs. However, this methodology did not allow the precise discrimination of microvesicles and exosomes (Figure 6D).

Finally, TEM analysis on isolated vesicles allowed the clear visualization of the vesicles surrounded by the lipid bilayer (Figure 6E). It underlined also the morphological and dimensional heterogeneity of the vesicles present in the sample, in line with TRPS data. The vesicles also appear sufficiently isolated with a low level of clustering in both samples.

### Biological Effect of hBMSC-EVs on hACs

**FIGURE 7:**
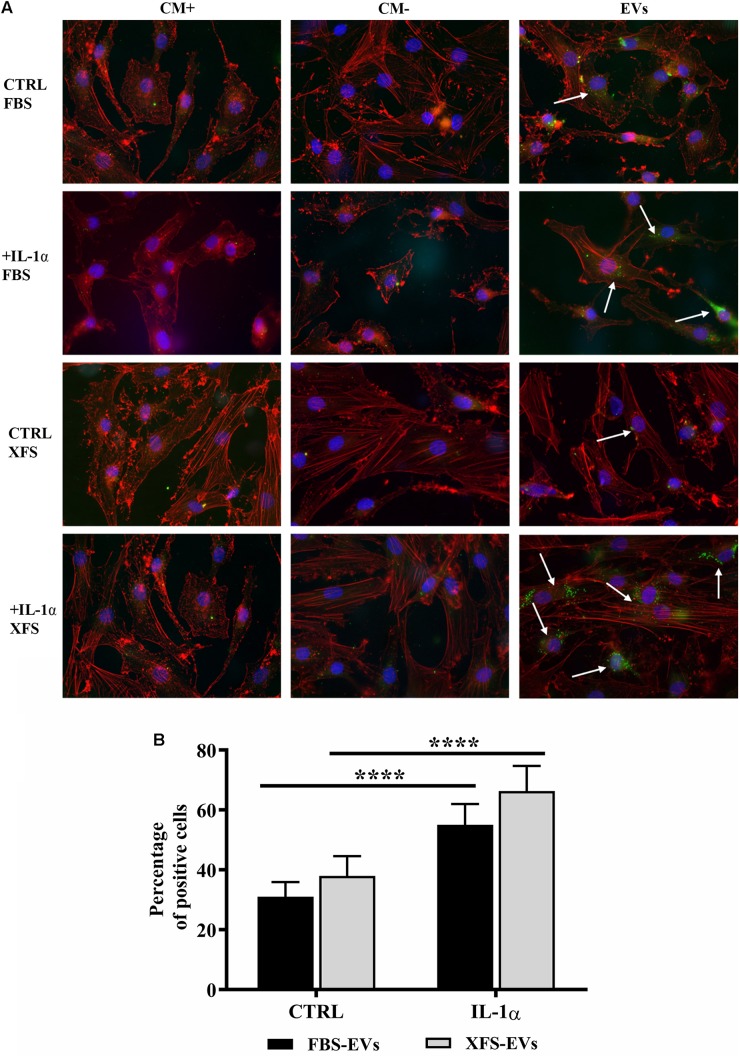
Uptake of EVs from hACs and OA hACs. **(A)** PKH67-stained hBMSC-EVs uptake by hACs and OA-hACs. **(B)** Data are represented as mean ± SD and are representative of five independent experiments. Quantification was performed on hACs and OA hACs pre-treated with IL-1α and then treated with PKH67-stained EVs. Positive cells were counted on at least five different areas for each experiment. ****P < 0.0001, two-way ANOVA.

BMSC-EVs were recently reported to mediate cartilage repair and regeneration (Cosenza et al., 2017; Vonk et al., 2018). To evaluate this potential, the interactions of EVs with recipient hACs were characterized. The potential of hACs and OA hACs to internalize both FBS-hBMSC-EVs and XFS-hBMSC-EVs were assessed post induction of an inflammatory process. hBMSC-EVs not only interacted but were taken up by OA-like chondrocytes (Figure 7A).

A quantitative analysis was also performed by counting the percentage of positive cells per region of interest (ROI, Figure 7B). FBS- and XFS-hBMSC-EVs showed a similar internalization efficiency both in normal (31.0 ± 4.8 and 37.9 ± 6.6%, respectively) and the inflammatory condition (55.0 ± 6.9% and 66.3 ± 8.3%), although EVs released by XFS-hBMSCs tended to be internalized with greater efficiency. Both types of EVs were internalized with higher efficiency following treatment with the interleukin. Although quantitative analysis did not show significant differences, the share of XFS-hBMSC-EVs per cell in OA-hAC appeared to be much higher and the signal was located close to the nucleus (Figure 7A).

### hBMSC-EVs Exhibited a Chondro-Protective Role

**FIGURE 8:**
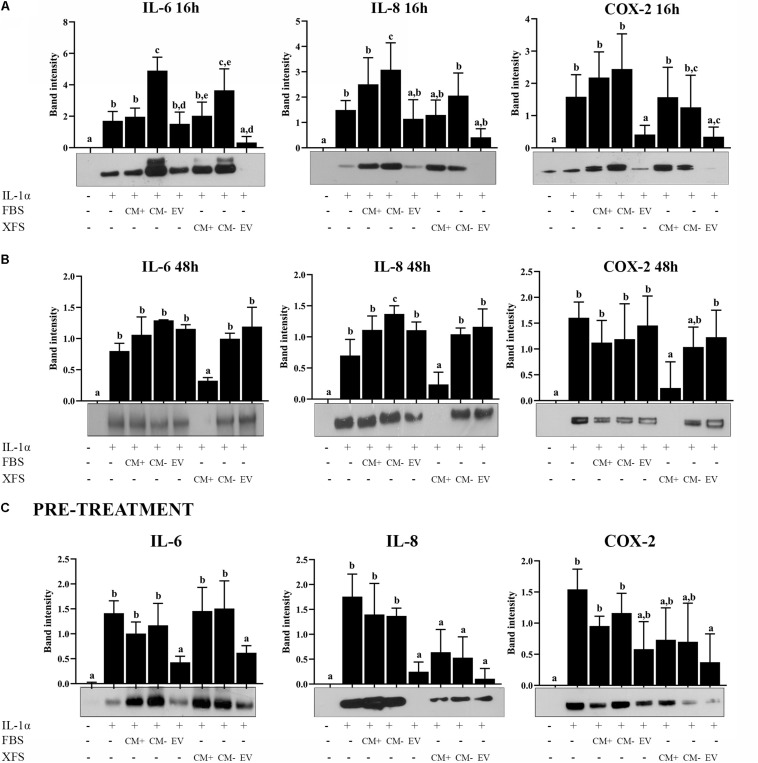
Response of OA-hACs to treatment with EVs. **(A)** Response of OA-hACs to 16 h hBMSC-EVs treatment in an inflammatory microenvironment with IL-1α. Western blot analysis of secreted IL-6 and IL-8 and COX-2 cellular content was performed, and the corresponding band intensity was determined by densitometric analysis. The intensity values were normalized to tubulin protein expression. Data are shown as mean ± SD of at least five independent experiments. Values with shared letters are not significantly different (*P* > 0.05) according with two-way ANOVA followed by Tukey as *post hoc* test. **(B)** Response of OA-hACs to 48 h hBMSC-EVs treatment in an inflammatory microenvironment with IL-1α. Data are shown as mean ± SD of at least five independent experiments. Values with shared letters are not significantly different (*P* > 0.05) according with two-way ANOVA followed by Tukey as *post hoc* test. **(C)** Response of hACs after 3 h pre-treatment with hBMSC-EVs and subsequent inflammatory induction with IL-1α for 16 h. Western blot analysis of secreted IL-6 and IL-8 and COX-2 cellular content. The intensity values were normalized to tubulin protein expression. Data are shown as mean ± SD of at least five independent experiments. Values with shared letters are not significantly different (*P* > 0.05) according with two-way ANOVA followed by Tukey as *post hoc* test.

To investigate whether hBMSC-EVs had the same anti-inflammatory effect as CM, confluent hACs were treated for 16 and 48 h with IL-1α ± 1 μg/ml FBS-hBMSC-EVs or XFS-hBMSC-EVs or a corresponding dose of FBS/XFS-CM^+^ or FBS/XFS-CM^–^. At variance with CM, modulation of IL-6, IL-8, or COX-2 after the treatment with FBS- or SF-hBMSC-EVs was observed after 16 h (Figure 8B). In particular, both types of EVs were able to blunt IL-8 secretion and COX-2 production after 16 h treatment. Only XFS-EVs determined a decrease of IL-6 to control levels. Completed and depleted media derived from both conditions were not able to revert the inflammatory condition after 16 h treatment (Figure 8A).

After 48 h, in line with the previous data, only XFS-CM^+^ produced the modulation of IL-6, IL-8, and COX-2, while the beneficial effect of both types of EVs was lost (Figure 8B).

In a parallel experiment, hACs were pre-treated for 3 h with 1 μg/ml FBS-hBMSC-EVs or 1 μg/ml XFS-hBMSC-EVs or a corresponding dose of FBS/XFS-CM^+^ or FBS/XFS-CM^–^. The inflammatory process was induced with treatment by IL-1α for 16 h. Protein levels of IL-6, IL-8, and COX-2 were measured and both types of EVs were shown to elicit a significant reduction in IL-6, IL-8, and COX-2 levels. Furthermore, an anti-inflammatory effect was achieved also by XFS-hBMSC-CM^+^ and -CM^–^, with a slight reversal of the increase in IL-8 and COX-2 induced by IL-1α (Figure 8C). Taken together, these data suggested that hBMSC-EVs could have a chondro-protective role and XFS culture enhances this therapeutic potential, that could be an assisted effect of EVs and soluble factors.

**FIGURE 9:**
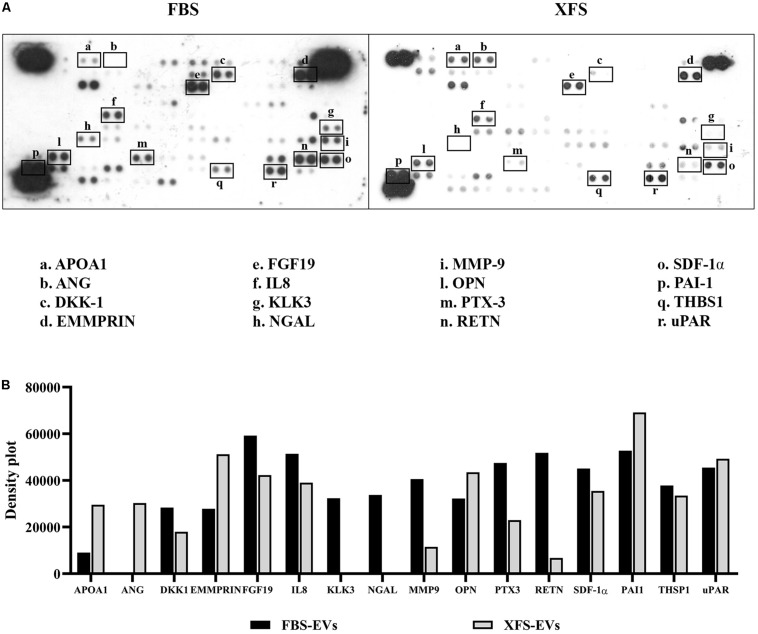
Characterization of protein profile of EVs derived from hBMSCs cultured in FBS- or XFS-containing medium. **(A)** Cytokine array membrane of hBMSCs-EVs (FBS-EVs and XFS-EVs) representative of a single experiment performed on a pool of EVs derived from three different primary cultures. **(B)** Quantification of the integrated pixel density for each identified cytokine using Fiji ImageJ. Data are shown as mean. APOA1: apolipoprotein A1; ANG: angiogenin; DKK-1: dickkopf-1 WNT signaling pathway inhibitor; FGF-19: fibroblast growth factors; IL8: interleukin-8; KLK3: Kallikrein 3; NGAL: lipocalin-2; MMP-9: metalloproteinase-9; OPN: osteopontin; PTX-3: pentraxin-3; RETN: resistin; SDF-1α: stromal cell derived factor 1α; PAI-1: serpin E1; THSP-1: thrombospondin-1; uPAR: plasminogen activator, urokinase.

To better understand the factors involved in the observed effects, we investigated the protein content of EVs. We performed the cytokine array on lysed EVs, to determine if they contained the same factors as the CM. The analysis of hBMSC-EVs showed both EV preparations contain proteins that has been founded in CM, such as pentraxin-3 (PTX-3), Dickoff-1 (DKK-1), thrombospondin-1 (TSP-1), osteopontin (OPN) and urokinase-type plasminogen activator receptor (uPAR). Moreover, we found other expressed proteins, such as Emmprin, FGF-19, IL-8, Serpin E1 (PAI1). Factors like Kallikrein 3 (KLK3), Lipocalin-2 (NGAL) and Metalloproteinase-9 (MMP9) were detected in FBS-hBMSC-EVs, while Apolipoprotein A1 (APOA1) and Angiogenin (ANG) were overexpressed in XFS-hBMSC-EVs (Figures 9A,B). Table 2 shows the ontologies related to the EVs proteins modulated by XFS, with the relatives *P*-values.

**TABLE 2:** Gene Ontology analysis of cytokine array on EVs.

	APOA1	ANG	DKK1	EMMPRIN	FGF19	IL8	KLK3	NGAL	MMP9	OPN	PTX3	RETN	SDFlα	PAI1	THSP1	uPAR	N^°^	P
Defense response	*	*				*	*	*		*	*	*	*	*	*	*	12	9.2E-8
Movement of cells or subcellular component	*	*		*	*	*			*			*	*	*	*	*	11	3.7E-7
Regulation of angiogenesis						*	*							*	*		4	9.5E-4
Cell proliferation	*	*			*	*			*			*	*		*		8	6.1E-4
Wound healing	*									*				*	*	*	5	2.2E-3
Regeneration	*									*			*				3	8.5E-3
Homeostatic process	*	*						*		*			*				5	4.2E-2

*Biological processes identified by cytokine clustering from 3 to 12 of the selected cytokines. APOA1: Apolipoprotein A1; ANG: angiogenin; DKK-1: dickkopf-1 WNT signaling pathway inhibitor; FGF-19: fibroblast growth factors; IL8: interleukin-8; KLK3: Kallikrein 3; NGAL: lipocalin-2; MMP-9: metalloproteinase-9; OPN: osteopontin; PTX-3: pentraxin-3; RETN: resistin; SDF-1α: stromal cell derived factor 1α; PAI-1: serpin E1; THSP-1: thrombospondin-1; uPAR: plasminogen activator, urokinase.*

## Discussion

In the last decade, the use of stem cells, and particularly MSCs, has increased considerably in many applications, including treatment of OA and bone defects in large animal models (Murphy et al., 2003) and in humans (Quarto et al., 2001; Vega et al., 2015). In most cases, an *in vitro* expansion of MSCs must be performed prior to their clinical application, with the choice of the optimal culture system an important challenge. In this scenario, the elaboration of animal-free culture media is particularly relevant for cell isolation and expansion for clinical purposes (Muraglia et al., 2017). The xeno-free environment is an important aspect to be considered to remove any source of variability and improve MSC acceptability in the context of regulatory guidelines.

In this study, we used a novel xeno- and serum-free culture medium to produce cells and cellular products ready for clinical usage, without any animal-associated contamination. hBMSCs isolated and cultured in XFS showed a higher and more stable proliferation rate than FBS-hBMSCs, suggesting that the XFS culture can enable the efficient production of therapeutic cells in a shorter time. These data are consistent with those reported for adipose-derived MSCs and human dental pulp stem cells cultured in chemically defined xeno-free media (Lee et al., 2017; Mochizuki and Nakahara, 2018). The maintenance of the typical MSC-phenotype according to the ISCT criteria (Dominici et al., 2006) underpins the use of this XFS culture system for MSC isolation. However, a noteworthy difference is represented by the corresponding decrease of CD106 expression on the cell surface. CD106, also known as Vascular Cell Adhesion Molecule-1 (VCAM-1), is an adhesion molecule which binds integrins, and its expression has been correlated with the differentiation potential of hBMSCs. For instance, CD106^+^-hBMSCs were found to be less osteogenic but more adipogenic than CD106^–^-hBMSCs (Fukiage et al., 2008). In line with this observation, we found a greater osteogenic potential of the XFS-hBMSCs (data not shown). It is important to note that XFS culture conditions also blunted HLA-DR expression. This is a key point given the involvement of this protein in activation of the immune system and the consequent rejection of allografts (Choo, 2007). Therefore, these results suggest that the hBMSCs grown in the xeno-free medium would not elicit an alloreactive response, making it an excellent candidate for use in clinical settings, for instance in MSC-based therapy for OA. Moreover, reduced apoptosis after growth in the serum/xeno-free medium was observed. Manufacturing of stem cells and progenitor cells for clinical use entails indeed an *in vitro* culture that could increase cell death. Our results showed that a xeno-free expansion could sustain the culture without loss in cell viability, as already described for induced pluripotent stem cells (McGrath et al., 2019).

In recent years, the therapeutic potential of MSCs has been widely investigated with extensive efforts by the scientific community focusing on elucidating their biological mechanisms of action in tissue repair and regeneration. While the original hypothesis underlying stem cell regenerative therapies was based on functional recovery as a consequence of stem cell differentiation (Bollini et al., 2013), it is now clear that other mechanisms of action play a critical role in these tissue rescue/repair processes. A recent paradigm shift has emerged suggesting that the biomolecules synthesized by stem cells may be as important, or even more so, than differentiation of the cells in eliciting functional tissue repair. In essence, an alternative mechanism has been proposed, the paracrine effect, where MSCs secrete biologically active molecules that exert beneficial effects on injured tissues. This hypothesis is supported by *in vitro* and *in vivo* studies showing that many cell types respond to paracrine signaling from MSCs, causing the modulation of a large number of cellular responses, such as survival, proliferation, migration and gene expression (Hocking and Gibran, 2010; Mancuso et al., 2019).

In our study, the characterization of secreted factors derived from the two populations revealed that hBMSCs grown in FBS-containing medium appeared richer in proteins like CHI3L1. This glycoprotein is involved in tissue remodeling, angiogenesis, cell survival and proliferation and is synthesized by several tissues of mesenchymal origin (Johansen, 2006).

On the other hand, hBMSCs grown in XFS-containing medium exhibited over-expression of several bioactive molecules involved in angiogenesis, tissue homeostasis and remodeling, immunomodulation and wound healing. Interestingly, we observed a three-fold increase of PTX3, an acute phase reactant whose regulatory role in inflammation (Deban et al., 2010) and wound healing or tissue remodeling has been widely described. Furthermore, it is well known that the inflammatory response following tissue damage induces PTX-3 production by MSCs (Bottazzi et al., 2010) and recent studies in murine models found that MSC-derived PTX-3 could play a role in promoting tissue repair (Cappuzzello et al., 2016). Therefore, the culture of hBMSCs in XFS may increase their potential for wound healing and tissue regeneration. Furthermore, an increase of Thrombospondin-1 (THBS1), a matricellular protein, was observed in XFS cultured cells compared to those grown in FBS-containing medium. THBS1 is known for its anti-angiogenic and anti-inflammatory effects as well as its anti-fibrogenic properties and a role in matrix preservation (Dobaczewski et al., 2010). Recently, a chondroprotective role has been described in some animal models of OA (Maumus et al., 2017) and rheumatoid arthritis (Manns et al., 2006; Rico et al., 2007). The data presented also showed a slight but statistically significant increase of factors, such as DKK-1 and osteopontin (OPN). DKK-1 is an antagonist of the Wnt signaling, that is implicated in bone and cartilage biology. It is well known that Wnt pathway is essential for cartilage homeostasis, however it has been reported that its excessive activation plays a crucial role in OA development and led to cartilage degradation, increasing chondrocytes hypertrophy and cartilage-degrading matrix metalloproteinases (MMPs) expression, while decreasing type II collagen production (Wang et al., 2019). Finally, OPN is a protein that interacts with several receptors, including integrins, giving rise to numerous functions such as cell adhesion, survival, migration and immune regulation (Wang and Denhardt, 2008). OPN is abundantly secreted by MSCs and implicated in their osteogenic differentiation (Rickard et al., 1994). Further investigation of the mechanisms of action of all these proteins using *in vitro* culture systems is necessary to understand the ultimate therapeutic benefits of the MSC secretome.

Our data suggest that hBMSCs grown in XFS could have a chondroprotective, anti-inflammatory effect and they could represent a potential therapeutic strategy for diseases such as OA and for bone repair, since they secreted bioactive molecules involved in these processes, as showed by the GO analysis performed. In order to validate our hypothesis, we studied the effect of CM derived from the two populations on hACs in an *in vitro* OA model described previously (Ulivi et al., 2008; Pereira et al., 2013), that uses IL-1α to mimic the inflammatory process that occurs following damage. In fact, when a lesion occurs in the joint, this pro-inflammatory cytokine is one of the most potent catabolic factors and is responsible for the induction of several mediators of cartilage degeneration such as IL-6 and IL-8. In the inflammatory milieu generated, cells adjacent to the injury site are not able to organize an appropriate regenerative response and consequently normal cartilage structure and composition are not restored.

We observed that the addition of CM derived from hBMSCs cultured in XFS-containing medium to hACs counteracted the IL-1α-induced secretion of the pro-inflammatory cytokines IL-6 and IL-8. However, this effect was not achieved by CM derived from hBMSCs cultured in FBS, where the inflammatory process was maintained and was not resolved. These results are in line with the results of the cytokine array data showing the upregulation of several proteins with anti-inflammatory properties, as mentioned above. We found that COX-2, an enzyme activated during the acute-phase response, was also significantly reduced in hAC extracts after XFS-CM treatment, but not after FBS-derived CM treatment. In cartilage, the activation of COX-2 has been described in the differentiated growth plate and also during inflammation (Langenbach et al., 1999). Ulivi et al. showed that in chondrocytes, COX-2 was expressed via p38/NF-kB activation and nuclear translocation during inflammatory response (Ulivi et al., 2008).

Inhibition of NF-kB has been recently reported in response to hBMSC-EVs where the EVs inhibited the translocation of NF-kB to the nucleus of primary human osteoarthritic chondrocytes (Vonk et al., 2018). Here, we extend these observations and reported that the novel culture system used produces cells able to secrete bioactive factors to the medium with the capability to restore the homeostatic status of chondrocytes after an inflammatory insult. After a short time (4 h) exposure to either FBS-hBMSC-CM or XFS-hBMSC-CM was not able to blunt the activation of NF-kB induced by IL-1α, while a longer treatment (40 h) caused a reduction of the NF-kB activity. This inhibitory effect was stronger in the case of XFS-hBMSC-CM given that even the lowest concentration of CM (1 μg/ml) was able to reduce nuclear NF-kB translocation to levels comparable to those observed in controls. Based on these data, we suggest that the activation and the subsequent inhibition of NF-kB is involved in the inflammation response and resolution controlled by the CM. NF-kB activation leads to the production of a number of pro-inflammatory factors including IL-6 and IL-8, as well as stress response genes including COX-2. Therefore, we could suggest that XFS-hBMSC-CM contains soluble factors and/or EVs able to inhibit NF-kB activation leading to a reduction of IL-6 and IL-8 release by OA-hACs. Because FBS-hBMSC-CM, despite its ability to inhibit NF-kB nuclear translocation is not able to downregulate IL-6 and IL-8, this release is a matter that deserves more investigation. In this context, our observation that hBMSC-CM in the presence of IL-1α was able to reduce not only NF-kB, but also COX-2 expression, indicates an early intervention to obtain the resolution of the inflammation process. Therefore, we propose that in cartilage the hBMSC-CM would promote a cascade of events leading to resolution of the IL-1α-induced inflammation.

This study focused on the paracrine functionality of hBMSCs cultured in a novel xeno-free culture system for clinical therapy in comparison to those in standard FBS culture conditions. Thereafter, we characterized EVs secreted by the cells in the two different culture conditions. We observed that culture with XFS allows the isolation of cells that secrete a higher amount of EVs, although the protein content and the expression of the surface markers CD105 and CD63 did not vary. A further advantage of XFS is the fact that, being a chemically defined culture medium, it lacks the vesicular contamination associated with serum. This allows a clean, safety and ready-to-use clinical-grade product to be obtained.

In line with the chondroprotective biomolecules found in XFS-hBMSCs-CM, it was observed that EVs derived from serum-free cultured cells tended to be more internalized by hACs than those derived from FBS-hBMSCs under basal conditions. Inflammation (i.e. IL-1α treatment) increased the internalization of FBS-hBMS-EVs and XFS-hBMSC-EVs to the same extent. Moreover, unlike what was observed with total CM, EVs are able to revert the upregulation of the levels of COX-2, IL-6, and IL-8 induced by IL-1α, but with a different timing. Interestingly, EVs seem to be effective already after a 16 h treatment. These different response times could be due to the fast EVs internalization (after 3 h they are taken up from hACs), that could justify a rapid mechanism of action. However, the beneficial effect does not last but is lost already after 48 h treatment. This aspect must be taken in consideration for a future *in vivo* application, as well as for clinical translation. Multiple administrations could be required to ensure a lasting effect over time.

Finally, the pre-treatment with FBS-hBMSC-EVs or XFS-hBMSC-EVs inhibited the pro-inflammatory effects of IL-1α. Interestingly, this particular effect was observed for both populations of EVs, but also when hACs were pre-treated with XFS-CM^+^ or XFS-CM^–^. This unique effect suggests that the chondroprotective effects of XFS-CM and EVs are borrowed from a dual effect of EVs and soluble factors. Differently, in the case of FBS-CM and EVs only the EVs seem to have a chondroprotective effect.

The cytokine profiling of EVs supports this hypothesis. Indeed, EVs are particularly rich of proteins overexpressed in XFS-hBMSC-CM that are involved in wound healing and immune response, such as DKK-1, THSP1, PTX-3 and uPAR. These factors are detectable in both preparations and they could be considered as important key factors in the observed effects.

Other interesting cytokines have been found. Particularly interesting is the overexpression, in XFS-hBMSC-EVs, of apolipoprotein A1 (APOA1). It is involved in defense response, cell proliferation, wound healing, homeostasis and regeneration, as emerged from GO. APOA1 is the main high-density lipoprotein. It plays a crucial role in the redistribution and clearance of cholesterol, regulating its efflux to flow into the liver. APOA1 has been shown to be expressed in cartilage during chondrocyte differentiation, both *in vivo* and *in vitro* (Gentili et al., 2005). Moreover, it has also been shown that an alteration in lipidic metabolism and APOA1 expression could be implicated in OA progression (Tsezou et al., 2010).

In both preparations of EVs we found considerably high levels of EMMPRIN and PAI1. The Extracellular Matrix Metalloproteinase Inducer (EMMPRIN) is a matrix metalloproteinase modulator and multifunctional cell recognition molecule. Its role is controversial. Some studies have correlated this molecule to the increase of MMPs production and the progression of joint destruction in RA and OA (Tomita et al., 2002; Zhu et al., 2006). However, EMMPRIN has been demonstrated to inhibit TNF-induced apoptosis and the NF-kB-dependent proinflammatory cytokines secretion in synovial tissue during RA (Zhai et al., 2016). Plasminogen activator inhibitor-1 (PAI-1) is an inhibitor of fibrinolysis. It is related to bone metabolism. Although it has been shown that PAI-1 deficiency accelerates subchondral osteopenia after induction of OA in mice, its role in OA remain unclear (Moritake et al., 2017).

Ultimately, in FBS-hBMSC-EVs we found NGAL, MMP-9 and RETN. Human neutrophil gelatinase–associated lipocalin (NGAL) was found to be associated to the MMP-9 activity in cartilage degradation, so it could be related to progressive cartilage loss in OA (Gupta et al., 2007). Resistin (RETN) is an adipocytokine associated with inflammation and insulin resistance. It has been shown that it could be play a role in OA pathogenesis. Indeed, RETN has been found in synovial fluid of OA patients and in OA cartilage. It has been also related to cartilage degradation by several studies (Lee et al., 2009; Koskinen et al., 2014). The presence of these factors may not be a benefit but could also be due to the donors. However, the inhibitory and chondroprotective effect of FBS-EVs seems to be the result of a positive balance in favor of pro-resolving and pro-restoration cytokines. In any case, the down regulation of these cytokines exerted by the culture in XFS represents a further advantage of this culture system, in view of a possible therapeutic application. Further studies and a deeper analysis are essential to better investigate the involvement and any effects of the detected cytokines.

In summary, FBS-hBMSC-CM was able to inhibit NF-kB nuclear translocation but did not affect protein levels of pro-inflammatory cytokines. In contrast, FBS-hBMSC-EVs showed a chondroprotective effect on hACs as previously reported (Vonk et al., 2018). XFS-hBMSC-CM and EVs exhibited a strong inhibitory effect on IL-1α-induced inflammation and a significant chondroprotective effect.

Taken together, the data presented indicated that XFS culture medium is a relevant tool to obtain clean and ready-to-use cells and cellular products, amenable for clinical treatment of diseases such as OA, given their chondroprotective properties, at least in an *in vitro* model. Further *ex vivo* or *in vivo* studies are needed to validate our observations.

## Data Availability Statement

The datasets generated for this study are available on request to the corresponding author.

## Author Contributions

CG, JM, and MP conceived and designed the study. MP, GS, SC, DR, LS, LP, DP, KC, and CG collected and/or analyzed the data. MP wrote the manuscript. CG, JM, and FB edited the manuscript.

## Conflict of Interest

The authors declare that the research was conducted in the absence of any commercial or financial relationships that could be construed as a potential conflict of interest.
