# Supplementary material for: The Secretome Derived From Mesenchymal Stromal Cells Cultured in a Xeno-Free Medium Promotes Human Cartilage Recovery in vitro
Source: Front Bioeng Biotechnol. 2020 Feb 14;8:90. doi: 10.3389/fbioe.2020.00090 (PMC7033421; doi:10.3389/fbioe.2020.00090)
Supplement: Supplementary file 1 [file Table_1.DOCX]

Supplementary Material

## Supplementary Figures


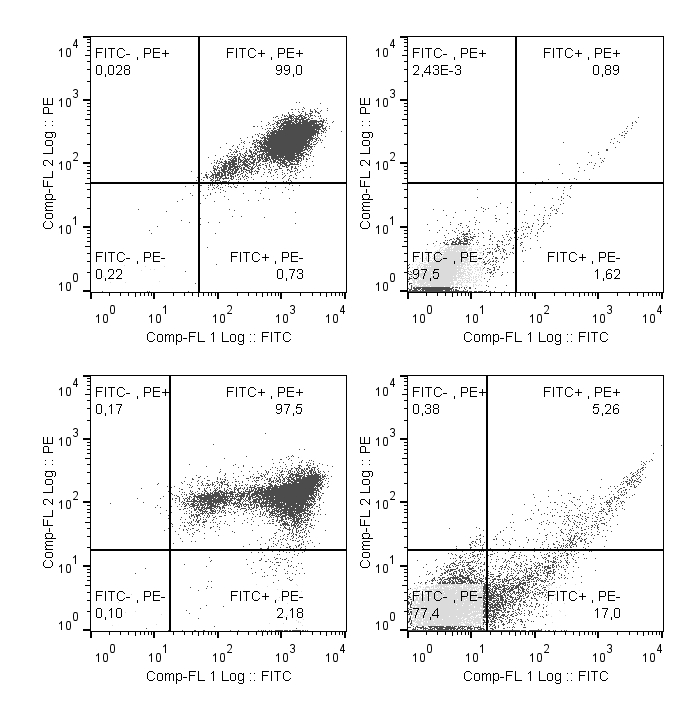
**Suppementary Figure 1. Representative flow cytometry analysis of FBS-hBMSCs (top panels) and XFS-hBMSCs (bottom panels) after 72 h of starvation.** Staining with annexin-V and PI is shown. An internal control with dead cells (10 min at 90 °C) was included to set the gates (left panels).


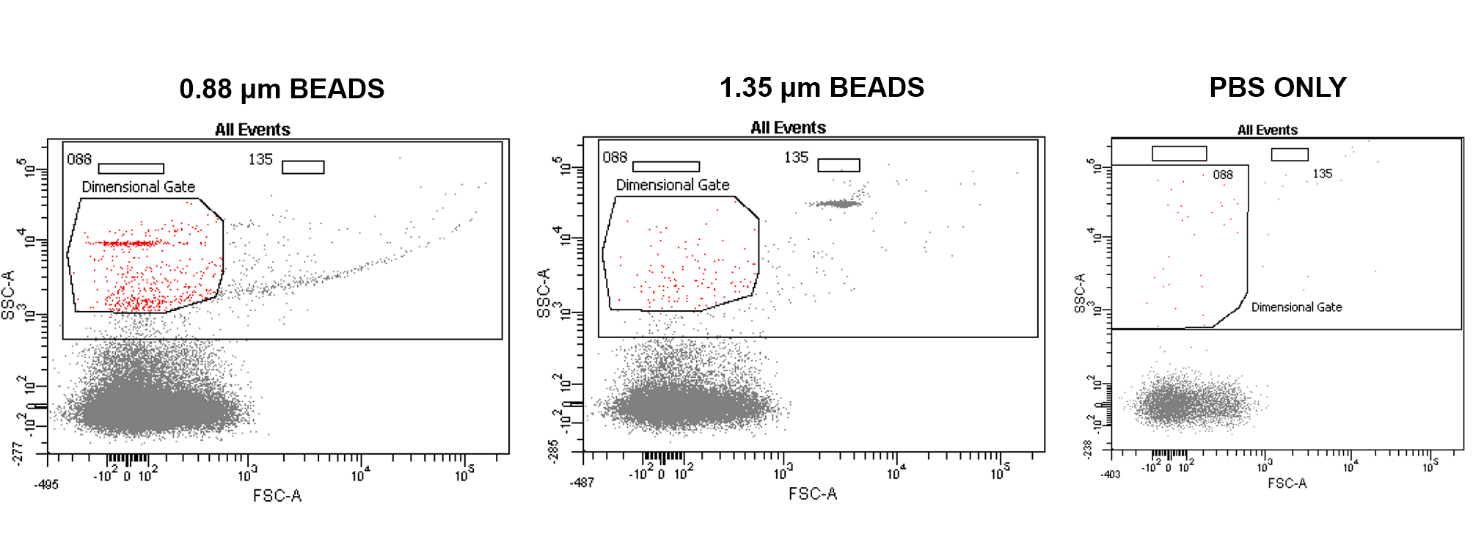


**Supplementary Figure 2. Set up and flow cytometry controls for EVs analysis.**
